# Comparison of Morning Heart Rate Variability at the Beginning and End of a Competition Season in Elite Speed Skaters

**DOI:** 10.3390/sports8120164

**Published:** 2020-12-14

**Authors:** Taro Iizuka, Michihiro Kon, Taketeru Maegawa, Jun Yuda, Toru Aoyanagi, Hideyuki Takahashi, Tomoaki Atomi, Miho Shimizu, Yoriko Atomi

**Affiliations:** 1 Material Health Science Laboratory, Graduate School of Engineering, Tokyo University of Agriculture and Technology, Tokyo 184-8588, Japan; t-iizuka@badminton.or.jp (T.I.); mshmz@cc.tuat.ac.jp (M.S.); 2 Nippon Badminton Association, Tokyo 160-0013, Japan; 3 Faculty of Liberal Arts and Sciences, Chukyo University, Nagoya 466-8666, Japan; kon.michihiro@gmail.com; 4 Faculty of Sports and Health Sciences, Fukui University of Technology, Fukui 910-8505, Japan; taketeru@topaz.ocn.ne.jp; 5 Faculty of Physical Education, Japan Women’s College of Physical Education, Tokyo 157-8565, Japan; jyuda@jwcpe.ac.jp; 6 Department of Sport Life Management, Nippon Sport Science University, Kanagawa 227-0033, Japan; aoyanagi@nittai.ac.jp; 7 Faculty of Health and Sport Sciences, University of Tsukuba, Ibaraki 305-8577, Japan; takahashi.hideyuk.ga@u.tsukuba.ac.jp; 8 Department of Physical Therapy, Faculty of Health Sciences, Kyorin University, Tokyo 181-8612, Japan; tatomi@ks.kyorin-u.ac.jp

**Keywords:** heart rate variability, speed skating, fatigue, autonomic nervous activity, measure of physiological fatigue in athletes, physiological fatigue measurement, physiological fatigue indices, elite athletes

## Abstract

The aim of this study was to clarify whether the physiological fatigue status of elite speed skaters is influenced by the approximately five-month international competition season by comparing morning heart rate variability (HRV) at the beginning of the competition season (Japan Single Distances Championships: JSDC) with that at the end of the competition season (World Single Distances Championships: WSDC). Five international-class speed skaters participated in the study. HRV indices and subjective fatigue were measured each morning of the four days prior to the first races of the JSDC and WSDC in the 2007/2008 season. The parasympathetic HRV indices: root mean square of the successive R-R interval differences (RMSSD) (JSDC, 61.0 ms; WSDC, 42.1 ms; *p* < 0.05), high-frequency component power (HF) (JSDC, 1393 ms^2^; WSDC, 443 ms^2^; *p* < 0.05), and normalized unit of HF (HFnu) (JSDC, 53.2%; WSDC, 25.5%; *p* < 0.05) were lower for the WSDC than for the JSDC. The decrease in these indices may reflect the skaters’ accumulated fatigue during the course of the competition season. Morning measurements of HRV may thus be an efficient way for elite speed skaters and coaches to objectively monitor physiological fatigue throughout the competition season.

## 1. Introduction

During the competition season, international-class elite speed skaters race in frequent competitions that take place in different countries over the course of approximately 5 months. International competitions are organized as a series of World Cup meets [1], in which speed skaters strive to consistently achieve superior results to earn a spot in major competitions, such as the Olympic Games and World Single Distances Championships (WSDC), scheduled at the end of the season. To be competitive in the major competitions at the end of the season, elite speed skaters and coaches face the difficult task of avoiding the accumulation of fatigue during the course of the demanding competition season. During the season, elite speed skaters are required to maintain their physical condition in a variety of training and food environments, in addition to coping with physiological stress from frequent long-haul travel and associated jet-lag that may negatively influence their physical condition [2,3]. When creating an effective seasonal conditioning plan, it is essential to precisely evaluate how the fatigue status of an elite speed skater is altered at the end of a competition season. However, the impact of a competition season on the fatigue status of elite speed skaters has not been objectively examined.

Measuring heart rate variability (HRV) is a noninvasive method for the evaluation of cardiac autonomic nervous activity [4,5]. Examination of HRV to evaluate cardiac parasympathetic nervous activity, alone or in combination with sympathetic nervous activity, is effective for objectively monitoring the fatigue status of athletes [5,6,7,8]. A decrease in parasympathetic HRV indices reflects an increased fatigue status in athletes [6,8,9,10,11,12,13]. Iizuka et al. [12] reported that changes in fatigue status of elite badminton players significantly correlated with changes in parasympathetic HRV indices. Naranjo et al. [13] reported lower parasympathetic HRV values and higher fatigue in professional soccer players during the final phase of the competition season. Therefore, monitoring parasympathetic HRV indices may provide an objective method for evaluating alterations in the fatigue status of international-class elite speed skaters.

The aim of this study was to investigate the impact of the competition season on the fatigue status of international-class elite speed skaters by measuring HRV indices. We compared HRV indices and subjective fatigue measured during precompetition periods for the first (Japan Single Distances Championships: JSDC) and last (World Single Distances Championships: WSDC) competition of the 2007/2008 season. We hypothesized that decreases in parasympathetic HRV indices would be observed during the precompetition period for the WSDC when compared to those for the JSDC.

## 2. Materials and Methods

### 2.1. Subjects

Five international-class speed skaters (three men and two women, mean age—22.8 ± 1.6 years) who represented Japan in the 2007/2008 competition season participated in this study. Of the eight speed skaters initially recruited for the measurements in JSDC, five speed skaters who could eventually compete in WSDC were included in the study. Before the investigation began, all of the skaters provided written informed consent to participate in the study. The study was reviewed and approved by the Tokyo University of Agriculture and Technology Ethics Committee (No.200603-0218) and conducted in accordance with the Declaration of Helsinki.

**Figure 1:**
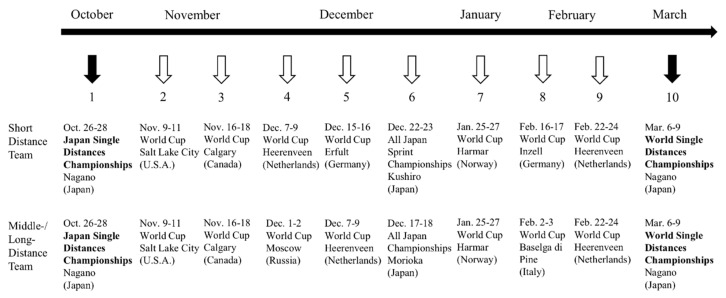
Competition schedule for the 2007/2008 speed skating season.

During the approximately five-month season, the skaters raced in eight international and two domestic competitions, which corresponds to one competition every two weeks in different countries (Figure 1). All of the skaters were ranked within the top 20 in the World Cup standings for the 2007/2008 season, and subsequently participated in the 2010 Vancouver Olympic Games.

In both the JSDC (October 2007) and the WSDC (March 2008), the skaters competed in multiple races. In both competitions, the first race for the four short-distance skaters was a 500-m race, and that for the one middle/long distance skater was a 1500-m race.

### 2.2. Procedures

The JSDC and WSDC, both of which took place at the same indoor ice-skating rink in Nagano, Japan, were important competitions for the skaters and their coaches. The JSDC was the first competition of the season, and the results would determine the speed skaters who would represent Japan in the 2007/2008 World Cup. The WSDC took place at the end of the 2007/2008 season, and the results would determine the world champion for each race distance.

**Figure 2:**
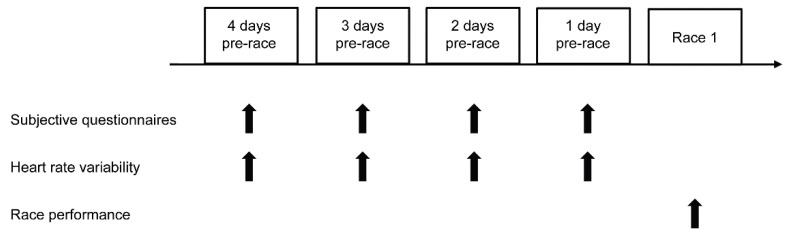
Data-collection schedule for the days prior to the first race (Race 1) of the Japan Single Distances Championships (JSDC) and the World Single Distances Championships (WSDC).

Before the competitions, the participants had training sessions with their respective company teams, and all the teams gathered in Nagano five days prior to the first races of each competition. Therefore, data were collected from the skaters each morning of the four days prior to the first races of each competition (Figure 2). Data collections were conducted at similar times of the day (around 7 a.m.) to avoid the influence of circadian variations on HRV. During the data collection periods, the skaters’ training loads were relatively low to reduce fatigue and help them prepare for the first race. The skaters were given a day off two days before the start of the competitions.

### 2.3. Measurements

#### 2.3.1. Subjective Fatigue

Subjective fatigue was measured using a 100-mm visual analog scale. The skaters were asked to rate their degrees of fatigue along a 100-mm line between 0 (no fatigue) and 100 (maximum fatigue) [14].

#### 2.3.2. HRV

Following a rest period of at least 1 min, the beat-to-beat heart rate (HR) of each skater was recorded in the seated position for 5 min using a Polar S810 heart rate monitor (Polar Electro Oy, Kempele, Finland) under controlled breathing of 15 breaths per minute [15]. Data processing was performed using Kubios HRV analysis software (Kubios HRV Standard, version 3.0.2., Kubios, Kuopio, Finland) [4,16]. For the time domain HRV indices, we obtained the standard deviation of all R-R intervals (SDNN) and the square root of the mean squared differences of successive R-R intervals (RMSSD). SDNN represents both the parasympathetic and sympathetic activity, whereas RMSSD represents parasympathetic cardiac modulation [4,5]. For the frequency domain HRV indices, we obtained the absolute power of the low-frequency (LF: 0.04–0.15 Hz) and high-frequency (HF: 0.15–0.40 Hz) band components. In addition, the normalized unit of HF (HFnu: HF/(LF + HF) × 100) and the LF/HF ratio were analyzed. HF reflects parasympathetic activity [17], and HFnu and the LF/HF ratio are used to evaluate the autonomic nervous system balance [4,17].

#### 2.3.3. Race Performance

Official race results were used to compare the skaters’ performances at the JSDC and WSDC. As the subjects competed in different events during the first race of each competition, individual race times in the WSDC were transformed to percentages of the race times in the JSDC, which allowed us to compare the race performances as a group for each competition.

### 2.4. Statistical Analysis

Data are presented as median and interquartile range. For all variables, data collected for four days before the start of each competition were averaged and used for further analyses. The Shapiro-Wilk test was used to examine normal distributions for all variables. Due to the non-normal distribution of the data, as well as the small number of study participants, the Wilcoxon signed-rank test was used to determine significant differences between the JSDC and WSDC values for each variable. Effect size was determined by the coefficient r [18], interpreted as follows: an r value of 0.1 was small, 0.3 was moderate, 0.5 was large, 0.7 was very large and 0.9 was extremely large [19]. The relationships between the changes in HRV indices and the change in subjective fatigue from JSDC and WSDC were examined by Spearman’s rank rho (*ρ*) correlation coefficient. Differences were considered statistically significant when *p* < 0.05. The SPSS version 19 (IBM, Armonk, NY, USA) was used for all statistical analyses.

## 3. Results

### 3.1. Subjective Fatigue

Table 1 shows the subjective fatigue in each morning prior to the first races of the JSDC and WSDC. Table 2 shows the differences in subjective fatigue between the JSDC and WSDC. There was no significant difference in subjective fatigue between the JSDC and WSDC (*p* = 0.500).

### 3.2. HRV

Table 3 shows the HRV indices in each morning prior to the first races of the JSDC and WSDC. Table 4 shows the differences in HRV indices between the JSDC and WSDC. Although the differences were not significant, HR values for the JSDC tended to be higher than those for the WSDC (*p* = 0.080). The RMSSD for the WSDC was significantly lower than that for the JSDC (*p* = 0.043), while no significant difference was observed for the SDNN (*p* = 0.225). HF and HFnu were also significantly lower for the WSDC than for the JSDC (*p* = 0.043 and *p* = 0.043, respectively). No significant differences were observed for LF and LF/HF (*p* = 0.893 and *p* = 0.138, respectively).

### 3.3. Correlation between Changes in Subjective Fatigue and Changes in HRV

**Figure 3:**
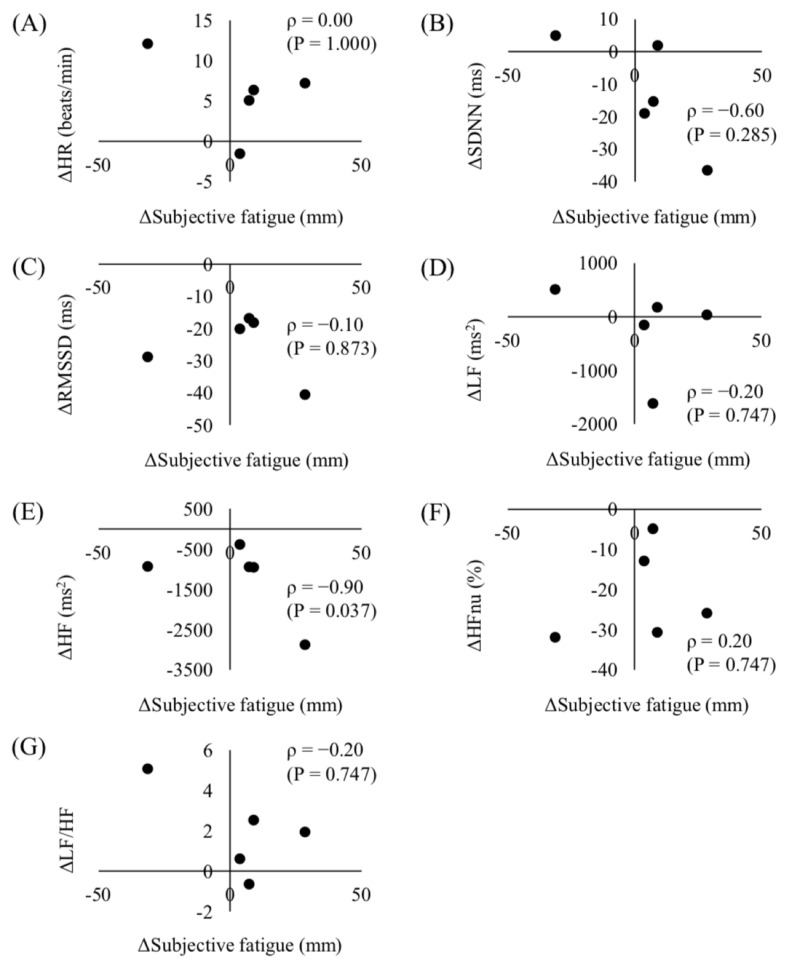
Correlation between the changes in subjective fatigue and the changes in heart rate variability (HRV) indices. (**A**) HR, (**B**) SDNN, (**C**) RMSSD, (**D**) LF, (**E**) HF, (**F**) HFnu, and (**G**) LF/HF from JSDC to WSDC.

Figure 3 shows the correlation between changes in subjective fatigue and changes in HRV indices from JSDC to WSDC. The change in subjective fatigue was significantly correlated with the change in HF (*p* = 0.037).

### 3.4. Race Performance

No significant differences were observed between race performances at the JSDC and WSDC (JSDC: 100.0% [100.0–100.0]; WSDC: 99.6% [99.4–100.1]; *p* = 0.225; r = 0.54).

## 4. Discussion

The aim of this study was to clarify whether the HRV of elite speed skaters at the end of the competition season (WSDC) was altered compared to that at the beginning of the season (JSDC). Although subjective fatigue and race performance values did not differ significantly between the competitions, we demonstrated that parasympathetic HRV indices, such as RMSSD, HF, and HFnu, were significantly decreased for the WSDC compared to those for the JSDC.

Previous HRV studies showed that a decrease in the parasympathetic activity is accompanied by accumulated fatigue in athletes [13,17,20,21]. Naranjo et al. [13] investigated the HRV indices of elite professional soccer players in a Spanish First Division club throughout the season and reported lower RMSSD values and higher fatigue because of the accumulation of player physical exertion from continuous matches during the final phase of the competition season. Likewise, in our study, the parasympathetic HRV indices were significantly decreased for the WSDC when compared to those for the JSDC, suggesting that the fatigue status of the skaters increased over the course of the competition season.

However, it should be noted that no significant change was observed in subjective fatigue. Crowcroft et al. [6] monitored HRV and subjective fatigue in competitive swimmers for 15 months and found that both measures were sensitive to changes in fatigue. Nevertheless, they suggested that the objective or subjective measures most useful for monitoring changes in fatigue may depend on the type of athlete. The authors also suggested that both objective and subjective monitoring of fatigue status should be considered. Rabbani et al. [22] indicated that it is unknown whether resting HRV and self-reported wellness measures are able to detect the same tendency/pattern. Therefore, to detect changes in the fatigue status of an elite speed skater, it may be worthwhile to measure subjective fatigue along with parasympathetic HRV indices.

Flatt et al. [10] suggested that parasympathetic HRV responses differ individually due to the differences in individual fitness and fatigue status. We demonstrated that the change in HF was significantly correlated with the change in subjective fatigue. Thus, our results suggest that HF measurements may be effective for objectively evaluating individual changes in the fatigue status of speed skaters. Monitoring individual changes in fatigue status by measuring HF, and modulating training load to recover in between competitions, would be effective for elite speed skaters to avoid accumulating fatigue during the course of the competition season.

Some studies have shown that decreased activity of the parasympathetic nervous system is associated with a decrease in athletic performance [11,21,23]. Garet et al. [23] examined the relationship between the HRV indices and competitive swimmers’ performance in a 400-m freestyle race and found that an individual’s worst performance was associated with his or her lowest parasympathetic activity, while their best performance was associated with his or her highest parasympathetic activity. However, as Flatt et al. [9] have pointed out, whether HRV is associated with anaerobic performance remains unknown because the majority of previous studies investigated the relationship between HRV and athletes’ endurance performance. In our study, four of the five skaters competed in sprint races, where anaerobic capacity is more involved in race performance. This may be why the WSDC race performance did not significantly decline despite significant decreases in parasympathetic HRV indices. Race performance in speed skating involves a variety of factors. Although both competitions took place at the same ice-skating rink, race performance could have been influenced by the development of skating skills during the competition season.

This study had several limitations. First, as we aimed to investigate the impact of the competition season on the HRV indices of elite speed skaters, we were only able to recruit five study participants. Further investigations are needed to examine the impact of the competition season on the HRV indices, fatigue status and race performance of elite speed skaters, and the study samples should include more middle/long distance speed skaters. Second, we could not monitor the skaters’ training load during the data-collection periods. Parasympathetic HRV indices have been shown to be altered by an increased training load [20,24,25,26,27,28]. Therefore, we cannot exclude the possibility that the significant decrease in the parasympathetic HRV indices for the WSDC was induced by a higher training load than that for the JSDC. However, the investigation was conducted during precompetition periods when training loads were kept relatively low in order to prepare for the competitions. Third, as we compared the HRV indices for the first and the last competitions of the season, we could not describe changes in the HRV indices over the course of the competition season. It is necessary to conduct more frequent monitoring of the HRV indices to further clarify how fatigue accumulates during the competition season in elite speed skaters.

In conclusion, in our study participants, the parasympathetic HRV indices were significantly lower for the WSDC than for the JSDC. To the best of our knowledge, this is the first study to practically suggest that the physiological fatigue status of international-class speed skaters is altered during the competition season. Monitoring parasympathetic HRV indices may be an efficient method for objectively evaluating changes in the fatigue status of international-class elite speed skaters. Morning HRV indices could be utilized to create effective plans to prevent fatigue accumulation among speed skaters during the competition season.

## Figures and Tables

**Table 1:** Description of subjective fatigue in each morning prior to the first races of the Japan Single Distances Championships (JSDC) and World Single Distances Championships (WSDC).

	Competition	4 Days Pre-Race	3 Days Pre-Race	2 Days Pre-Race	1 Day Pre-Race
Subjective fatigue (mm)	JSDC	50.0 (41.0–50.0)	50.0 (50.0–50.0)	33.0 (23.0–49.0)	39.0 (1.0–50.0)
	WSDC	50.0 (22.0–67.0)	30.0 (15.0–50.0)	50.0 (38.0–50.0)	50.0 (38.0–50.0)

Data are presented as median and interquartile range.

**Table 2:** Comparisons of subjective fatigue in the Japan Single Distances Championships (JSDC) and World Single Distances Championships (WSDC).

	**JSDC**	**WSDC**	***p*** **-Value**	Effect Size
Subjective fatigue (mm)	46.3 (25.0–47.0)	50.0 (32.0–53.5)	0.500	0.30

Data are presented as median and interquartile range.

**Table 3:** Description of heart rate variability indices in each morning prior to the first races of the Japan Single Distances Championships (JSDC) and World Single Distances Championships (WSDC).

Index	Competition	4 Days Pre-Race	3 Days Pre-Race	2 Days Pre-Race	1 Day Pre-Race
HR (beats/min)	JSDC	54.5 (52.4–55.5)	51.1 (49.2–54.2)	51.6 (50.1–52.1)	52.4 (51.8–55.6)
	WSDC	55.1 (55.1–57.7)	56.0 (50.3–60.8)	61.8 (55.2–63.2)	59.7 (57.9–64.9)
SDNN (ms)	JSDC	67.0 (66.7–82.6)	94.9 (62.4–111.5)	63.8 (63.1–115.8)	79.6 (57.7–109.1)
	WSDC	88.2 (83.5–97.0)	76.7 (40.0–79.0)	73.0 (55.1–75.3)	64.3 (58.4–73.7)
RMSSD (ms)	JSDC	48.7 (48.5–51.5)	74.7 (73.4–84.9)	67.4 (58.5–97.0)	54.7 (40.7–75.8)
	WSDC	60.5 (45.6–83.0)	38.2 (27.0–45.5)	36.8 (36.8–46.4)	33.9 (27.5–40.6)
LF (ms^2^)	JSDC	1312 (762–3152)	1690 (1016–2872)	2735 (1443–3178)	1706 (1089–2789)
	WSDC	2328 (2088–2490)	866 (611–1491)	1473 (1411–3021)	1101 (1079–1378)
HF (ms^2^)	JSDC	831 (758–1034)	2048 (1509–3367)	1931 (1015–3176)	951 (426–1908)
	WSDC	958 (582–2311)	435 (236–499)	424 (315–828)	268 (172–272)
HFnu (%)	JSDC	42.5 (38.8–45.3)	53.9 (53.3–54.3)	47.0 (41.3–58.8)	56.9 (35.8–63.7)
	WSDC	31.4 (17.2–49.8)	27.9 (27.4–28.8)	16.8 (14.3–37.8)	19.5 (16.5–29.5)
LF/HF	JSDC	1.35 (1.21–1.58)	0.85 (0.84–0.87)	1.13 (0.70–1.42)	0.76 (0.57–1.79)
	WSDC	2.18 (1.01–4.81)	2.59 (2.47–2.65)	4.95 (1.65–6.00)	4.12 (2.38–5.07)

Data are presented as median and interquartile range.

**Table 4:** Comparisons of heart rate variability indices between the Japan Single Distances Championships (JSDC) and World Single Distances Championships (WSDC).

Index	JSDC	WSDC	*p*-Value	Effect Size
HR (beats/min)	52.6 (51.8–52.8)	60.0 (54.6–62.5)	0.080	0.78
SDNN (ms)	76.1 (75.5–79.1)	71.1 (63.7–81.0)	0.225	0.54
RMSSD (ms)	61.0 (60.3–62.2)	42.1 (38.0–42.1)	0.043	0.91
LF (ms^2^)	1326 (1226–2912)	1504 (1297–1736)	0.893	0.06
HF (ms^2^)	1393 (1339–1450)	443 (400–1064)	0.043	0.91
HFnu (%)	53.2 (49.4–56.2)	25.5 (21.8–30.9)	0.043	0.91
LF/HF	1.00 (0.81–1.16)	3.35 (2.75–4.26)	0.138	0.66

Data are presented as median and interquartile range.
